# Triptonide Effectively Inhibits Wnt/β-Catenin Signaling via C-terminal Transactivation Domain of β-catenin

**DOI:** 10.1038/srep32779

**Published:** 2016-09-06

**Authors:** Jessica Chinison, Jose S. Aguilar, Alan Avalos, Ying Huang, Zhijun Wang, D. Joshua Cameron, Jijun Hao

**Affiliations:** 1 College of Veterinary Medicine, Western University of Health Sciences, Pomona, CA 91766, USA; 2 College of Pharmacy, Western University of Health Sciences, Pomona, CA 91766, USA; 3 College of Optometry, Western University of Health Sciences, Pomona, CA 91766, USA; 4 Graduate College of Biomedical Sciences, Western University of Health Sciences, Pomona, CA 91766, USA

## Abstract

Abnormal activation of canonical Wnt/β-catenin signaling is implicated in many diseases including cancer. As a result, therapeutic agents that disrupt this signaling pathway have been highly sought after. Triptonide is a key bioactive small molecule identified in a traditional Chinese medicine named *Tripterygium wilfordii* Hook F., and it has a broad spectrum of biological functions. Here we show that triptonide can effectively inhibit canonical Wnt/β-catenin signaling by targeting the downstream C-terminal transcription domain of β-catenin or a nuclear component associated with β-catenin. In addition, triptonide treatment robustly rescued the zebrafish “eyeless” phenotype induced by GSK-3β antagonist 6-bromoindirubin-30-oxime (BIO) for Wnt signaling activation during embryonic gastrulation. Finally, triptonide effectively induced apoptosis of Wnt-dependent cancer cells, supporting the therapeutic potential of triptonide.

Canonical Wnt/β-catenin signaling plays important roles in embryogenesis and tissue homeostasis^1^. In the absence of Wnt ligands, the key regulator of this signaling pathway, β-catenin, is phosphorylated and subjected to proteolytic degradation by the destruction complex, Axin/GSK-3β/APC, in the cytoplasm. In the presence of Wnt ligands, however, the destruction complex is disassembled, and cytoplasmic β-catenin is stabilized then subsequently translocated to the nucleus where it interacts with transcriptional activators to initiate transcription of Wnt target genes. In addition, β-catenin, independent of its involvement in canonical Wnt signaling, also acts as a structural component of cell–cell adherent junctions, where β-catenin is tightly bound to E-cadherin^2^. Since aberrant Wnt/β-catenin signaling is associated with developmental malformations and many types of disease including cancer^3^,^4^,^5^, significant endeavors have been made to develop therapeutic reagents like small molecules to target this pathway^5^.

Triptolide and triptonide are two key bioactive small molecules identified in a traditional Chinese medicine named *Tripterygium wilfordii* Hook F. (also known as the Thunder God Vine or *lei gong teng*)^6^. They are structurally similar and only differ in one chemical group at position 14, a carbonyl group in triptonide and a hydroxyl group in triptolide (Fig. 1A,B). Both triptolide and triptonide have been known to have a broad spectrum of biological functions such as immunosuppression, anti-inflammatory, anti-fertility and neuroprotective effects^7^,^8^,^9^. In recent years, several studies of intracellular targets of triptolide have been reported^10^,^11^. For instance, Corson and colleagues have identified dCTP pyrophosphatase 1 (DCTPP1) as the biophysical target of triptolide by using a pull-down approach with a biotinylated photoaffinity derivative of triptolide. However, substantially higher concentrations of triptolide are required to bind and inhibit recombinant DCTPP1 than those reported for its biological effects, suggesting that DCTPP1 is unlikely to be the physiological target of triptolide^12^. Recently, triptolide was shown to attenuate aberrant activation of Wnt/β-catenin signaling pathway in cervical cancer cells by degrading β-catenin protein^13^. However, the cellular target of triptonide has been poorly studied. Here we show that triptonide can effectively inhibit canonical Wnt/β-catenin signaling by targeting the C-terminal transcription domain of β-catenin or its associated nuclear component, a mechanism different from triptolide.

## Results

### Triptonide effectively inhibits Wnt/β-catenin signaling through a mechanism different from triptolide

**Figure 1:**
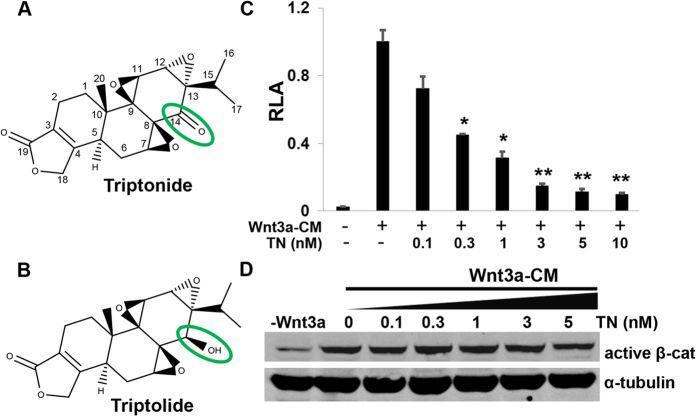
Triptonide effectively inhibits Wnt/β-catenin signaling through a mechanism different from triptolide. (**A**) Chemical structures of Triptonide and (**B**) troptolide are displayed. (**C**) Triptonide (TN in the figure) effectively inhibits Wnt/β-catenin signaling induced by Wnt3a conditional media (Wnt3a-CM) in STF293 cells that were stably transfected with TOPFLASH luciferase plasmid. The data was represented as mean relative luciferase activities (RLA) + SEM (n = 3). All the P values are compared to the luciferase activity induced by Wnt3a-CM (*P < 0.05; **P < 0.01). (**D**) Western blotting showed that triptonide did not attenuate Wnt3a-CM-induced active β-catenin expression.

**Figure 2:**
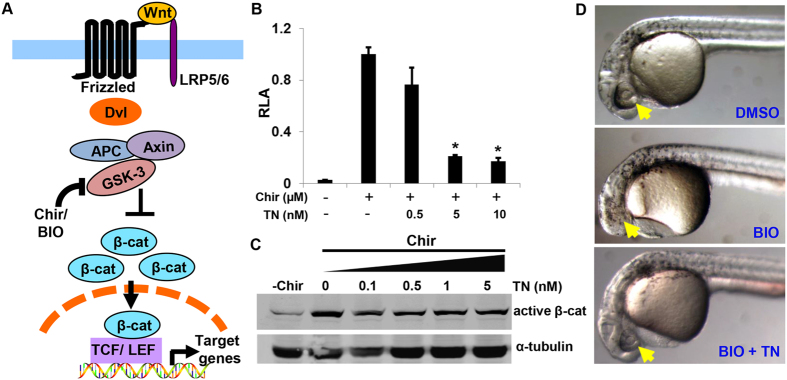
Triptonide may inhibit Wnt/β-catenin signaling downstream of GSK-3β, a key component of the β-catenin destruction complex. (**A**) Cartoon model illustrates Wnt/β-catenin signaling with various means to modulate the pathway. Briefly, disruption of the β-catenin degradation complex, through pharmacological inhibition of GSK-3β using the small molecule CHIR 99021 (Chir) or BIO, leads to nuclear β-catenin accumulation and subsequent Wnt reporter activation. (**B**) Triptonide blocked Wnt/β-catenin signaling induced by GSK-3β antagonist Chir (2 μM) in a dose dependent manner. Results of luciferase assay were represented as mean relative luciferase activities (RLA) + SEM (n = 3). All the P values are compared to the luciferase activity induced by Chir (*P < 0.05). (**C**) Triptonide did not regulate active β-catenin expression levels. (**D**) Triptonide reproducibly rescued the loss of eyes in in BIO-induced zebrafish embryos. Embryos were exposed to DMSO or 100 nM triptonide in the presence of 0.3 μM BIO at the shield stage (6 hpf) and the images were taken at 30 hpf. 15 embryos treated with 0.3 μM BIO resulted in 14 fish with double eye loss in the control, and 15 embryos treated with both 100 nM triptonide and 0.3 μM BIO led to 13 fish with appearance of both eyes at 30 hpf (P < 0.0001). P-value was calculated with GraphPad Prism V6.

Triptonide’s structure is closely related to triptolide which was previously reported as an inhibitor of Wnt/β-catenin signaling^13^ (Fig. 1A,B). Here, we examined whether triptonide can actually inhibit Wnt/β-catenin signaling in a way similar to triptolide. To measure the canonical Wnt/β-catenin signaling activity, luciferase assays were carried out in STF293 cells that are stably transfected with TOPFLASH-firefly luciferase reporter plasmid^14^,^15^. Our study showed that triptonide effectively inhibited Wnt3a (CM)-induced TOPFLASH-luciferase activity in a dose dependent manner with an IC_50_ of appropriately 0.3 nM, and triptonide alone had no effects on TOPFLASH-luciferase activity (Fig. 1C and Supplementary Figure 2), indicating that triptonide, like triptolide (Supplementary Figure 1A), is an inhibitor of Wnt/β-catenin signaling. In addition, RT-PCR was conducted to examine an impact of triptonide on expression of Wnt target genes, Axin2 and Cyclin D1. The result indicated that triptonide dramatically attenuated gene expression of Axin2 and Cyclin D1 induced by CHIR 99021 (Chir), a selective GSK-3β inhibitor which induces Wnt signaling by blocking β-catenin phosphorylation for subsequent degradation (Fig. 2A and Supplementary Figure 3).

As Wnt3a-CM induces disassembly of the cytoplasmic destruction complex Axin/GSK-3β/APC, GSK-3β is no longer capable of phosphorylating β-catenin for subsequent degradation^16^, resulting in active non-phosphorylated β-catenin which subsequently translocates to the cell nucleus. Previously triptolide was reported to attenuate Wnt3a-CM-induced β-catenin protein levels in a dose dependent manner^13^,^17^. We examined whether triptonide has a similar effect on β-catenin levels using the active non-phosphorylated β-catenin antibody. Interestingly, different from triptolide (Supplementary Figure 1B), triptonide did not downregulate Wnt3a-CM-induced β-catenin levels in HEK293 cells (Fig. 1D), suggesting that triptonide inhibits Wnt/β-catenin signaling through a mechanism different from triptolide.

To localize the cellular target of triptonide in the Wnt pathway, we treated STF293 cells with the selective GSK-3β inhibitor Chir alone or combinations with triptonide at various concentrations. Chir treatment alone robustly induced β-catenin dependent luciferase activity, and the Chir-induced TOPFLASH-luciferase activity was inhibited by triptonide in a dose-dependent manner (Fig. 2B). Furthermore Western blotting demonstrated that triptonide did not affect Chir-induced β-catenin levels in HEK293 cells (Fig. 2C). These results suggest that triptonide may target the β-catenin destruction complex or a signaling component lying downstream of the destruction complex.

### Triptonide rescues the “eyeless” phenotype induced by GSK3 inhibitor BIO

The ectopically activated Wnt/β-catenin signaling during gastrulation is known to lead to an “eyeless” phenotype in zebrafish embryos^18^,^19^,^20^. Pharmacological inhibition of GSK3 with GSK3 inhibitor BIO copies the “eyeless” phenotype^15^,^21^,^22^. We examined whether triptonide can rescue the BIO-induced “eyeless” phenotype. Our data indicated that zebrafish embryos treated by BIO from 6 hour post fertilization (hpf) to 24 hpf resulted in the “eyeless” phenotype and triptonide reproducibly restored the eye development (Fig. 2D). This *in vivo* study confirmed that triptonide targets either a downstream component of the β-catenin destruction complex or the destruction complex.

### Triptonide does not block β-catenin translocation into the cell nucleus

**Figure 3:**
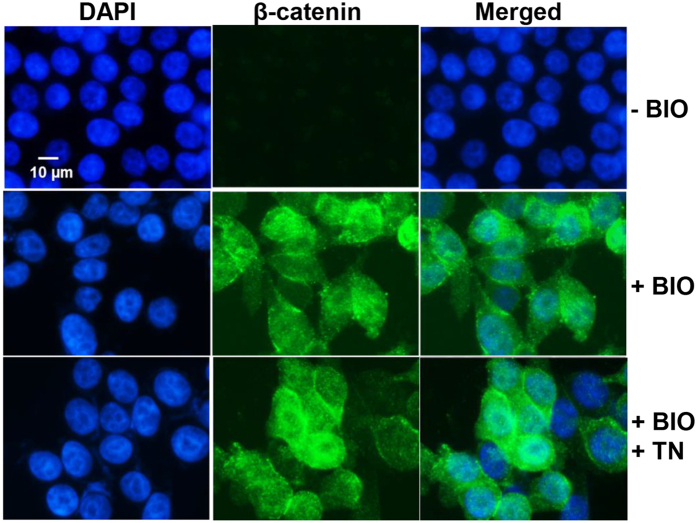
Triptonide does not block β-catenin nuclear translocation. Human colon carcinoma RKO cells were treated with DMSO, 2 μM BIO and a combination of 2 μM BIO with 5 nM triptonide respectively. After 24-hour incubation, the cells were immunostained for β-catenin (green) and counterstained with DAPI (blue).

**Figure 4:**
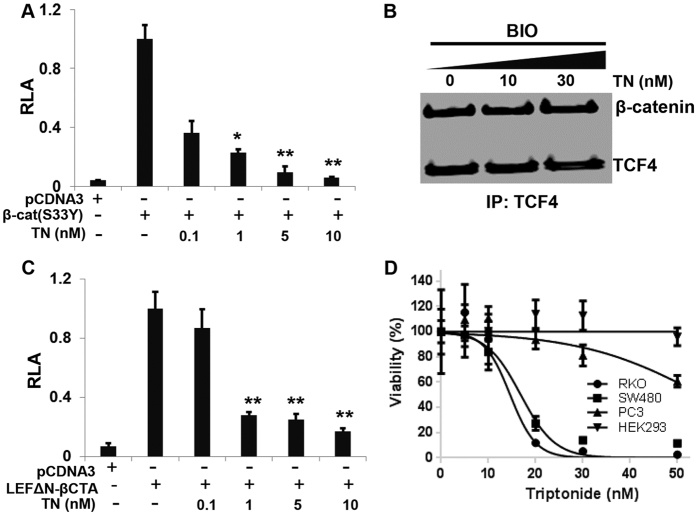
Triptonide blocks Wnt/β-catenin signaling presumably via the c-terminal transactivation domain of β-catenin, and promoted apoptosis in Wnt-dependent cancer cells. (**A**) Triptonide inhibited Wnt signaling induced by overexpression of the constitutively active human β-catenin mutant (S33Y) in TOPFLASH-luciferase assay in STF293 cells. The data was represented as mean percentage + SEM (n = 3), and all the P values are compared to the luciferase activity induced by the β-catenin mutant (S33Y) plasmid transfection (*P *< *0.05; **P *< *0.01). (**B**) Triptonide did not block the interaction between TCF4 and β-catenin. HEK293 cells were incubated with 2 μM BIO and increasing concentrations (0, 10 and 30 nM) of triptonide for overnight following by immunoprecipitation with anti-TCF4 and immunoblotting with anti-β-catenin and anti-TCF4. (**C**) Triptonide inhibited Wnt signaling induced by overexpression of the LEFΔN-βCTA in TOPFLASH-luciferase assay in STF293 cells. The data was represented as mean percentage + SEM (n = 3), and all the P values are compared to the luciferase activity induced by the LEFΔN-βCTA plasmid transfection (**P < 0.01). (D) Percentage of viable human cancer cells (SW480, RKO, DU145 and PC3), as determined by cell titer assays, following 72 hour treatment with increasing concentrations of triptonide. Results were from two independent experiments conducted each in triplicate, and the data was represented as mean percentage + SEM.

Human colon carcinoma RKO cells, which contain intact Wnt signaling components, exhibit very low E-cadherin expression on the cell surface and therefore lack the plasma-membrane-associated pool of β-catenin^23^. Utilizing this unique quality, we induced β-catenin expression in RKO cells with GSK-3 antagonist BIO and examined if triptonide could block cytoplasmic β-catenin translocation into cell nuclei. β-catenin expression at the basal condition (without BIO treatment) was hardly observed due to its phosphorylation and subsequent degradation (Fig. 3). However, BIO treatment which inhibits GSK-3 activity for β-catenin phosphorylation and degradation dramatically induced β-catenin translocation into nuclei (Fig. 3). Nevertheless, triptonide did not block and degrade the BIO-induced β-catenin in the cell nucleus (Fig. 3). To further verify our finding, we transiently transfected STF293 cells with the constitutively active human β-catenin mutant (S33Y) which is resistant to GSK-3β-mediated degradation due to the mutation of Serine 33, the GSK-3β phosphorylation site in β-catenin^24^, and then examined whether triptonide could inhibit this mutant-induced TOPFLASH-luciferase. Indeed, triptonide effectively abrogated the luciferase activity induced by the constitutively active human β-catenin mutant (S33Y) (Fig. 4A). Taken together, these results demonstrated that triptonide does not block β-catenin nuclear translocation and likely targets a downstream nucleic signaling component in the Wnt pathway.

### Triptonide targets a downstream nucleic component of Wnt/β-catenin signaling

β-catenin contains three domains: a N-terminal domain, a central 12-armadillo-repeat (AMR) domain and a C-terminal transactivation domain^25^. Numerous cofactors in the nucleus have been shown to interact with the β-catenin domains to regulate Wnt signaling^26^,^27^,^28^. It could be possible that triptonide may target β-catenin or some of these nucleic cofactors to disrupt their interactions with β-catenin for Wnt signaling suppression. Since T cell factor (TCF) is a critical transcription factor interacting with the β-catenin central AMR domain for Wnt signaling modulation^29^, we first examined if triptonide could block the interaction between TCF and β-catenin. HEK293 cells were incubated with triptonide or DMSO as a vehicle control, in the presence of 2 μM BIO overnight followed by immunoprecipitation with anti-TCF4 antibody and immunoblotting with anti-β-catenin and anti-TCF4 antibodies (Fig. 4B). The result showed that triptonide did not block the interaction between TCF4 and β-catenin AMR domain. As the N-terminal region of β-catenin is mainly involved in β-catenin degradation by binding of TrCP1 (also known as β-TrCP) E3 ubiquitin ligase, and triptonide did not regulate β-catenin levels for Wnt signaling inhibition (Figs 1D, 2C and 3), implying that triptonide unlikely targets the N-terminal region of β-catenin. Conversely, the C-terminal domain is a strong transactivator for Wnt signaling in the nucleus^30^, so we focused on examining whether triptonide disrupts the interaction between the C-terminal transactivation domain of β-catenin (βCTA) and its associated co-factors. Previous studies have shown that the LEFΔN-βCTA construct, a fusion of the βCTA domain (amino acids 695–781) with the TCF/LEF1 DNA-binding domain, is capable of stimulating a TCF/β-catenin responsive promoter^31^. We, therefore, transiently transfected the STF293 cells with LEFΔN-βCTA expression plasmid, followed by triptonide treatments at various concentrations. Compared to transfection with the control plasmid pCDNA3, the LEFΔN-βCTA dramatically induced TOPFLASH luciferase activity and triptonide effectively attenuated the LEFΔN-βCTA-induced luciferase activity in a dose-dependent manner (Fig. 4C), suggesting that triptonide specifically targets βCTA domain or its associated nuclear co-factor for the Wnt signaling inhibition.

### Triptonide promotes apoptosis in Wnt-dependent cancer cells

Abnormal activation of canonical Wnt/β-catenin signaling is implicated in many types of cancer^32^,^33^. Unlike its analog triptolide that has been extensively studied for its anticancer abilities^13^,^17^,^34^,^35^,^36^, no studies of anti-cancer properties of triptonide have been reported to date. We, therefore, evaluated the therapeutic potential of triptonide in the known Wnt signaling-dependent cancer cell lines including human colon cancer cell lines SW480 and RKO as well as prostate cancer cell lines PC3^24^,^37^,^38^. HEK293 cells were used as a control. After 72 hours of treatment, triptonide selectively kill these colon cancer cells with IC_50_ values of 14.6 nM and 16.8 nM for RKO and SW480 cells, respectively (Fig. 4D). Additionally, 50 nM triptonide, after 72 hours of treatment, decreased the PC3 cell viability at approximately 40% whereas it did not display killing activity in the control HEK293 cells (Fig. 4D). To confirm triptonide’s induction of apoptosis in the Wnt-dependent cancer cells, we used the CellEvent™ caspase-3/7 green detection reagent (ThemoFisher), a fluorogenic substrate for activated caspase-3/7 to perform an apoptosis assay^39^. Both HEK293 cells and SW480 cells were cultured in the 8-well Lab-Tek™ II Chamber Slide™ System (ThermoFisher) and treated with 20 nM triptonide or DMSO vehicle for 24 hours. The cell culturing medium which may contain some dead cells of SW480 was washed away and the only adherent cells were then subjected to the CellEvent™ caspase-3/7 green detection for apoptosis assay. In consistence to the cell viability assay, the result showed that triptonide induced apoptosis in SW480 cells, but not in the control HEK293 cells (Supplementary Figure 4). These results indicate that triptonide may be a potential agent for cancer therapeutics by targeting the Wnt/β-catenin pathway.

## Discussion

Here we reported that triptonide can effectively inhibit canonical Wnt/β-catenin signaling presumptively by targeting β-catenin transaction domain or its associated nuclear co-factor for Wnt signaling inhibition, a mechanism different from triptolide. We showed that triptonide dose-dependently inhibited Wnt/β-catenin signaling induced by Wnt ligand Wnt3a and GSK-3β inhibitor Chir. Additionally, triptonide did not block β-catenin’s nucleic translocation and its interaction with TCF4. We further narrowed down the potential target of tripotnide to the C-terminal domain of β-catenin-associated cofactors. Nevertheless, numerous co-factors are known to be associated with the βCTA including transcriptional mediator complexes, histone methyltransferases (such as mixed lineage leukemia protein), histone acetlytransferases (such as p300/CBP), chromatin modification BRG1, polymerase associated factor complexes, etc. It is also possible that the target of triptonide is a new factor which has not been identified in Wnt signaling before. To further identify triptonide’s target, a number of approaches may be considered including affinity-based proteomics (“pulldown”), two-dimensional gel electrophoresis, drug affinity responsive target stability (DARTS) and *in vivo* chip analysis, etc^40^.

It is interesting that triptolide and triptonide only differ in one chemical group at position 14, a hydroxyl group in triptolide and a carbonyl group in triptonide, but they display distinct molecular mechanisms. This could be because this single chemical difference substantially impacts the 3-dimension structures of the two compounds. The hydroxyl group in triptolide has a sp3 carbon which can form a tetrahedral structure while the carbonyl group in triptonide has a sp2 carbon which forms trigonal planar structure^41^. Thus, the oxygen atom in the carbonyl group of triptonide, which may serve as a critical hydrogen bond acceptor, is facing a different direction from the oxygen atom in the hydroxyl group of triptolide. This spatial structural change may lead to an alteration of binding profiles and activities of the two compounds.

Aberrant activation of canonical Wnt signaling is implicated in many types of disease, particularly responsible for over 90% of colorectal cancers^42^. Despite significant research efforts, to date, unfortunately no FDA approved drugs are currently available to block this pathway. *Tripterygium wilfordii* Hook F. extracts have already been used to treat rheumatoid arthritis and other diseases in China for decades, suggesting that its bioactive component triptonide may exhibit ideal drug-like properties^6^. This concept is further supported by the fact that triptonide effectively rescued the “eyeless” phenotype *in vivo* as a result of Wnt signaling activation in zebrafish. Finally, we showed that triptonide effectively induced apoptosis in cancer cells in which Wnt signaling is known to be aberrantly activated, but had no impact on the normal HEK293 cells. Triptonide that targets a very downstream of the Wnt signaling in the nucleus and exhibits potential desired drug properties may represent a therapeutic advantage as an anti-cancer agent.

## Materials and Methods

### Cell cultures and Wnt3a-conditioned media

HEK293 (human embryonic kidney), STF293 (HEK293 cells stably transfected with TOPFLASH-firefly luciferase reporter), PC3 (human prostatic adenocarcinoma), SW480 (human colon adenocarcinoma) and RKO (human colon carcinoma) cells were cultured in DMEM supplemented with 10% Fetal bovine serum FBS (Gibco, Grand Island, NY, USA) and 1% penicillin-streptomycin (Invitrogen, Carlsbad, CA). Cultures were maintained in a humidified incubator at 37 °C in 5% CO_2_. Wnt3a-conditioned media (Wnt3a-CM) was made from Wnt3A cell line (ATCC^®^ CRL2647™) according to instructions of the American Type Culture Collection (Manassas, VA), USA.

### Maintenance of Zebrafish

Zebrafish AB/TL wild-type strain was maintained under standard conditions at 28.5 °C on a 10-hours dark and 14-hours light cycle. Fertilized eggs were obtained by mating adult fish soon after the light was turned on. Embryos were staged according to hours postfertilization (hpf) and morphological criteria. All animal husbandry and experiments were approved and conducted in accordance with guidelines set forth by the Institutional Animal Care and Use Committee of Western University of Health Sciences.

### Transfections and Luciferase reporter assays

Transient transfection was performed using Fugene HD transfection reagent (Promega) according to manufacturer’s instructions. Briefly, 1 × 10^5^ STF293 cells were seeded in 96-well plates to culture in the growth medium without antibiotics^14^. After overnight culture, the cells were transfected with 0.1 μg plasmid (control plasmid pcDNA3 or constitutively active β-catenin^**S33Y**^, LEFΔN-βCTA or LEFΔN-VP16) with 0.05 μg renilla luciferase reporter pRL-TK plasmid, followed by small molecules’ treatment. After 18 ~ 24 hours incubation, the cells were then lysed, and cell extracts were subjected to dual luciferase assay (Promega). For luciferase reporter assays without transfection, STF293 cell lysates were subjected to Steady-Glo luciferase assay (Promega) according to manufacturer’s instructions, and the results were then normalized to cell titer, as determined using Cell Titer-Glo luminescence assay (Promega).

### Western blotting

Cells were lysed with RIPA buffer (Sigma) supplemented with protein inhibitors (complete ULTRA Tablets, Roche) and phosphatase inhibitors (PhosSTOP, Roche). The cell lysates were separated by 10% SDS-PAGE gels and transferred to a PDVF membrane (Millipore). The membrane was blocked with Odyssey Blocking solution (Li-Cor Biosciences) for 1 hour at room temperature, followed by incubation with primary antibodies at 4 °C overnight. The membrane was then washed with PBS with 0.1% Tween-20 before 1-hour incubation with secondary antibodies at room temperature. The primary antibodies used here included rabbit mAb active β-catenin (1:1000 dilution, Cell Signaling Technology) and mouse mAb α-tubulin (1:1000 dilution, Cell Signaling Technology). The secondary antibodies were IRDye 800CW Goat Anti-Mouse IgG (1:5000 dilution, Li-Cor) and IRDye 680RD Goat Anti-Rabbit IgG (1:5000 dilution, Li-Cor). The intensities of the bands were obtained using an Odyssey scanner and analyzed with Image Studio Ver 2.0.

### Co-immunoprecipitation

Cells were lysed in RIPA buffer (Sigma) supplemented with protease inhibitors (complete ULTRA Tablets, Roche) and phosphatase inhibitors (PhosSTOP, Roche). Cell lysate was incubated with the anti-rabbit TCF4 (Cell Signaling Technology, 1:100 dilution) at 4 °C for 2 hours followed by antibody-antigen conjugation with Protein A agarose beads (Santa Cruz Biotechnology) overnight according to the manufacturer’s instruction. After three cold PBS washes, the beads were spun down, and bound protein was eluted in LDS buffer (Invitrogen). Eluted protein was resolved in SDS-PAGE and transferred onto nitrocellulose membrane for Western blot analysis.

### Immunostaining of β-catenin translocation

Human colon carcinoma RKO cells cultured in 8-well chamber slides (Lab-Tek II) were treated with 5 nM triptolide or triptonide in the presence 2 μM 6-bromoindirubin-30-oxime (BIO) for 24 hours. The cells were fixed with 4% formaldehyde and then permeabilized with 0.2% Triton X-100 in PBS for 15 min at RT. The cells were incubated with blocking buffer (0.1% Tween-20, 1 mg/ml BSA, PBS) for 30 min at RT, followed by primary antibody staining with rabbit mAb active β-catenin (1:500 dilution, Cell Signaling Technology) in blocking buffer for overnight at 4 °C. After PBS washing, the cells were then incubated with Alexa Fluor 488 (1:1000, dilution, Molecular Probes) conjugated secondary antibodies for 1 hour at room temperature. After washing and DAPI staining, immunostaining images were taken using fluorescence microscopy (EVOS FL, Thermo Fisher Scientific).

### Cancer cell viability assay

Cancer cells (SW480, RKO and PC3) and HEK293 cells were seeded into 96-well plates at density of 1 × 10^4^ cells per well. After overnight incubation, triptonide was then added to the cells at varying concentrations, and the cells were incubated for an additional 72 hours followed by a cell viability assay using the Cell Titer-Glo luminescence assay (Promega) as per manufacturer’s recommendations.

### Real-time PCR (RT-PCR)

RNA was extracted by re-suspending and re-pipetting the cells in Lysis Buffer and purified by filtration following the manufacturer’s protocol (GeneJET RNA Purification Kit, Thermo Scientific). The first-strand cDNAs were synthesized using the SupeScript III kit (Invitrogen) according to the manufacturer’s instructions. Using the cDNA as a template, RT-PCR reactions were carried out using Fast SyberGreen (2x) Master Mix (Applied Biosystems). Reactions were performed in triplicate with a STEP ONE PLUS cycler (Applied Biosystems). Human GAPDH gene was used as an internal control. The following primer sets were used: human GAPDH: GGTGTGAACCATGAGAAGTATGA (forward), GAGTCCTTC CACGATACCAAAG (reverse); Axin2: Forward CTTATCGTGTGGGCAGTAAGA (forward), GTTCTCGGGAAATGAGGTAGAG (reverse). Cyclin D1: GGGTTGTGCTACAGATGATAGAG (forward), AGACGCCTCCTTTG TGTTAAT (reverse).

### Cell apoptosis assay

CellEvent™ caspase-3/7 green detection reagent (Themo Fisher) was used for cell apoptosis assay. In brief, after 24 hour treatment, the cell culturing media were replaced with 5% FBS in PBS buffer supplemented with a final concentration of 3 μM caspase-3/7 green detection reagent. After 1-hour incubation, the cells were then fixed with 3.7% formaldehyde for 15 minutes at room temperature, followed by another 10 minutes of incubation with 0.2% Triton X-100 in PBS buffer at 4 °C. Then 1μg/mL DAPI was added for counterstaining. The images were taken by using a fluorescence microscopy with absorption/emission maxima of ~502/530 nm (EVOS FL, Thermo Fisher Scientific).

### Phenotypic rescue of “eyeless” phenotype in zebrafish embryos

Embryos collected from crosses of AB wild type zebrafish were exposed to triptonide or DMSO in the presence of 0.3 μM BIO at the shield stage. After 18-hour incubation at 28.5 °C, the small molecules were washed out and the images were taken at 30 hpf. Restoration of eye defect as scored by presence of both eyes.

## Additional Information

**How to cite this article**: Chinison, J. *et al*. Triptonide Effectively Inhibits Wnt/β-Catenin Signaling via C-terminal Transactivation Domain of β-catenin. *Sci. Rep.* **6**, 32779; doi: 10.1038/srep32779 (2016).

## Supplementary Material

Supplementary Information
