# Supplementary material for: Triptonide Effectively Inhibits Wnt/β-Catenin Signaling via C-terminal Transactivation Domain of β-catenin
Source: Sci Rep. 2016 Sep 6;6:32779. doi: 10.1038/srep32779 (PMC5011721; doi:10.1038/srep32779)

Triptonide Effectively Inhibits Wnt/β-Catenin Signaling via C-terminal Transactivation Domain of β-catenin

Jessica Chinison[1*], Jose S Aguilar[1*], Alan Avalos[1], Ying Huang[2], Zhijun Wang[2], Joshua Cameron[3] and Jijun Hao[1,4 §]

[1] College of Veterinary Medicine, Western University of Health Sciences, Pomona, CA 91766, USA

[2] College of Pharmacy, Western University of Health Sciences, Pomona, CA 91766, USA

[3] College of Optometry, Western University of Health Sciences, Pomona, CA 91766, USA

[4] Graduate College of Biomedical Sciences, Western University of Health Sciences, Pomona, CA 91766, USA

* These authors contributed equally to this work.

§ Corresponding author: Jijun Hao, College of Veterinary Medicine, Western University of Health Sciences, Pomona, CA 91766. Phone: (909) 469-8686, Fax: 909-469-5635, E-mail: jhao@westernu.edu

Supplementary Figure 1: Triptolide inhibits Wnt/β-catenin signaling by degrading β-catenin. (A) Triptolide (TL in the figure) effectively inhibits TOPFLASH luciferase induced by Wnt3a-CM in STF293 cells. The data was represented as mean relative luciferase activities (RLA) + SEM (n=3). All the P values are compared to the luciferase activity induced by Wnt3a-CM (*P <0.05; **P <0.01). (B) Western blotting showed that triptolide significantly attenuated the β-catenin protein expression level.

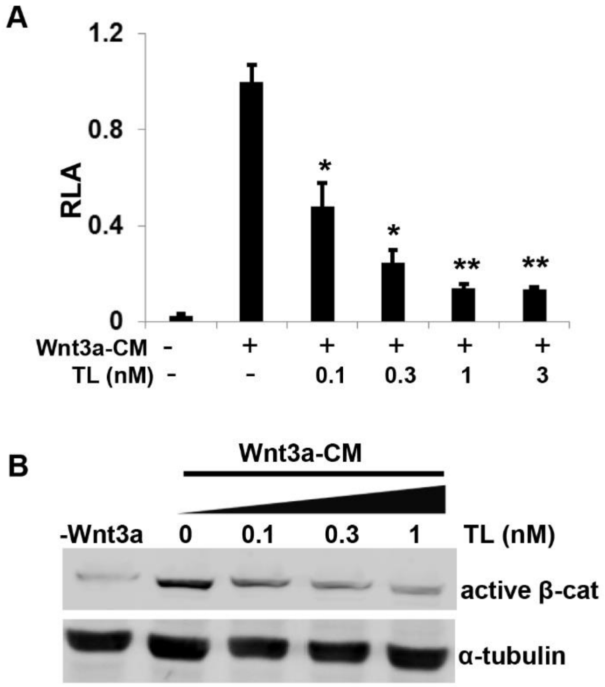

Supplementary Figure 2: Triptonide treatment alone does not alter Wnt/β-catenin signaling.

Triptonide alone had no effects on TOPFLASH-luciferase in STF293 cells. Values are reported as mean RLA + SEM.

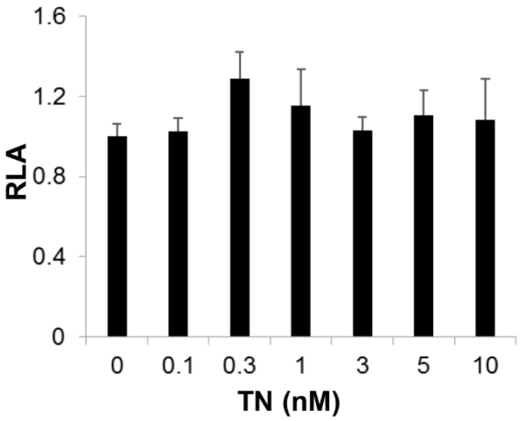

Supplementary Figure 3: Triptonide downregulates Wnt target gene Axin2 and Cyclin D1 RNA expression. In comparison to DMSO treated control (Ct) samples, 5 nM triptonide (TN) attenuated RNA expression of both Axin2 (A) and Cyclin D1 (B) induced by 2 µM Chir in STF293 cells. The RNA expression of the control samples was set to 1, and relative RNA expression level was calculated by normalizing Axin2 and Cyclin D1 RNA expression with the DMSO treated controls.

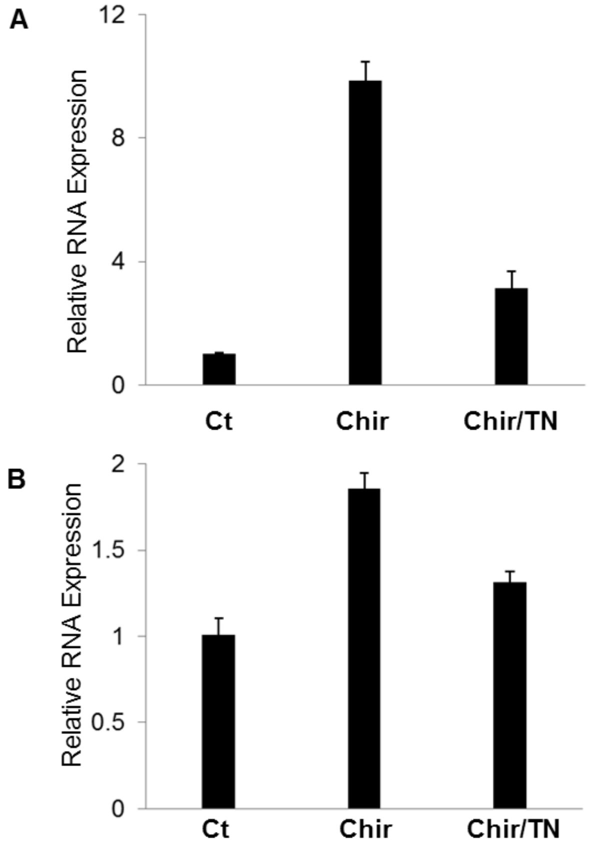

Supplementary Figure 4: Cell apoptosis assay in HEK293 cells and SW480 cells following treatment with DMSO, or 20 nM triptonide for 24 hours. The apoptotic cells that still adhered to chamber slides were detected with the CellEvent Caspase-3/7 Green Detection Reagent (ThermoFisher, yellow arrows) and DAPI was used for counterstaining.

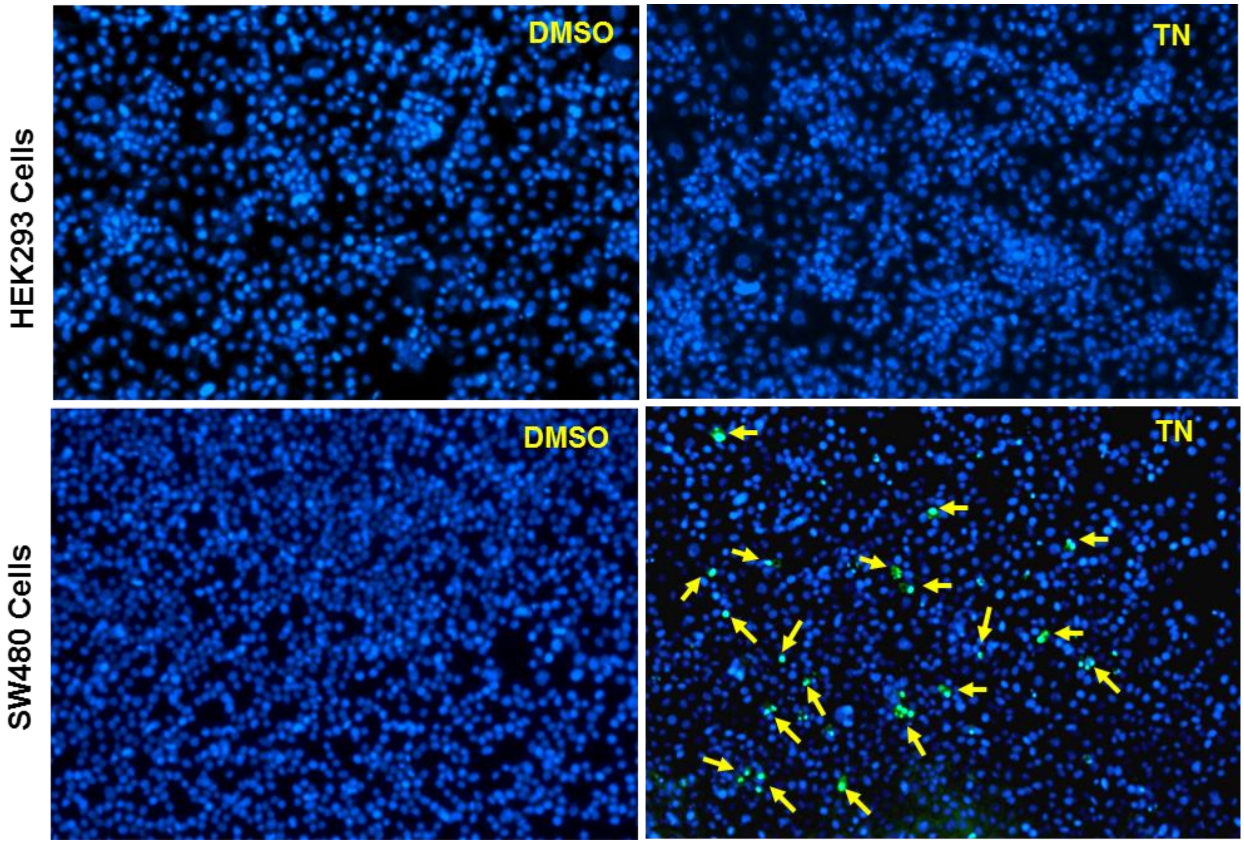

Supplement: Supplementary Information [file srep32779-s1.pdf]
